# A new species of the genus *Orthotemnus* Wollaston, 1873 (Coleoptera, Curculionidae, Cossoninae) from China

**DOI:** 10.3897/zookeys.472.8033

**Published:** 2015-01-19

**Authors:** Youssef Mohamed Omar, Runzhi Zhang, Steven R. Davis

**Affiliations:** 1 Plant Protection Department, Faculty of Agriculture, Assiut University, Assiut, EGYPT; 2 Institute of Zoology, Chinese Academy of Sciences, 1 Beichen West Road, Chaoyang District, Beijing 100101, China; 3 State Key Laboratory of IMPIR of China, 1 Beichen West Road, Chaoyang District, Beijing 100101, China; 4 Division of Entomology, Natural History Museum, and Department of Ecology & Evolutionary Biology. 1501 Crestline Drive-Suite #140 University of Kansas Lawrence, Kansas 66049-4401, USA

**Keywords:** Weevil, Proecini, morphology, systematics, plectra, key, China, new record

## Abstract

A new species, *Orthotemnus longitarsus* **sp. n.**, is described from China, representing the first record of the genus *Orthotemnus* Wollaston, 1873 from China. Illustrations of diagnostic features of the new species and a key to all six species of the genus *Orthotemnus* (including *Orthotemnus reflexus* Wollaston, *Orthotemnus disparilis* Pascoe, *Orthotemnus filiformis* Champion, *Orthotemnus expansus* Hustache and *Orthotemnus ulmi* Zherichin) are provided. All type specimens of the new species are deposited in the National Zoological Museum in the Institute of Zoology, Chinese Academy of Sciences, Beijing, China.

## Introduction

Based on the classification of Alonso-Zarazaga and Lyal (1999), *Orthotemnus* belongs to the tribe Proecini in the subfamily Cossoninae. The genus was erected by Wollaston (1873) for *Orthotemnus reflexus*, described from specimens collected by Wallace in Dorey, New Guinea, as well as in Batchian, Makian and Ceram. Pascoe (1885) described a second species, *Orthotemnus disparilis*, from Cape York, Northern Australia, and Champion (1914) a third, *Orthotemnus filiformis*, from a single specimen (♀ ?) collected by Scott at Silhouette in the Seychelles. The fourth species, *Orthotemnus expansus*, was described by Hustache (1955) based on one specimen collected from Watsa, Kibali-Ituri, D.R. Congo. Zherikhin (in Zherikhin and Egorov 1990) added the fifth species, *Orthotemnus ulmi*, collected from rotten timbers of elm (*Ulmus*) at the Ussuriysk reserve in the Far East of the USSR. Morimoto (1973) and Zhang (1992) presented diagnostic features for the genus *Orthotemnus* in their respective keys to the Oriental and Chinese genera of Cossoninae (also see Folwaczny 1973).

The aim of this paper is to establish the first record of the genus *Orthotemnus* for China by describing a new species from Jiangsu province in eastern China and to provide a key to all the known species currently recognised in *Orthotemnus*.

## Materials and methods

The type specimens of the new species are deposited at the Institute of Zoology (IOZ), Chinese Academy of Sciences (CAS), in Beijing, China. Morphological observations were made using a Zeiss Semi stereomicroscope Discovery V12, and photos were taken using a Micropublisher 5.0 RTV digital camera, model MP5.0-RTV-CLR-10A-color 10 BIT, attached to the same stereomicroscope. The recognition of the new species and the key to the species are based on comparison of the Chinese specimens with the original descriptions and illustrations of the previously described species.

All measurements were taken using an ocular micrometer. Abbreviations of characters given in the text are as follows: ACL – antennal club length; ACW – antennal club width; AFL – antennal funicle length; AL – antennal length; ASL – antennal scape length; BL – body length (excluding rostrum); EL – elytral length; EWB – elytral width at base; EWW – elytral width at widest part; PL – pronotal length; PW – pronotal width (widest part); RL – rostral length; RWA – rostral width at apex; RWB – rostral width at base.

Hind wing terminology follows Zherikhin and Gratshev (1995).

## Taxonomic treatment

### Orthotemnus

Taxon classificationAnimaliaColeopteraCurculionidae

Wollaston, 1873

#### Type species.

*Orthotemnus reflexus* Wollaston, 1873: 489, by monotypy.

#### Diagnosis.

*Body* (5.50–6.60 mm) not or slightly compressed longitudinally, elongate; derm glossy, glabrous, at most with sparse setae on elytra. *Rostrum* rather long and robust, much shorter than pronotum; longer than head; often wider before antennal insertion (equal width throughout: Wollaston (1873)). *Antennae* inserted above middle of rostrum in lateral view so that anterior part of scrobes visible from above; scapes reaching or passing hind margin of eyes, longer than funicles; funicles with seven segments. *Head* with forehead between eyes as broad as or broader than base of rostrum; postocular constriction not touching eyes. *Eyes* large, distance between eyes and postocular constriction shorter than diameter of eye. *Pronotum* not bisinuate at base (if weakly bisinuate, tarsal segment 5 broadest near base and tapering distally, or derm matt); triangular, truncate at base, as wide as elytral base. *Elytra* evenly parallel-sided, linearly truncated at base, not setose, recurved at their apex. *Legs*. with pro- and mesocoxae very widely and subequally separated, metacoxae less remote than pro- and mesocoxae; procoxae not very close to hind margin of prosternum; tibiae normal; tarsal groove of front tibiae not or bluntly pointed laterally; tarsi with segment 3 small, simple, entire or slightly notched, segment 5 normal, clavate, not compressed (Wollaston 1873, Hustache 1924, Morimoto 1973, Zhang 1992).

### Orthotemnus longitarsus

Taxon classificationAnimaliaColeopteraCurculionidae

Omar & Zhang sp. n.

http://zoobank.org/F8FA4CA4-ECE4-4998-8137-FA8A1EE5AB5C

Figs 1–4 , 5–11 , 12–18 , 19–22 , 23–31 , 32–37 , 38–40

#### Diagnosis.

Rostrum widening after antennal insertion; scapes passing posterior margin of eye and reaching postocular constriction; temples swollen; forehead with short median furrow; sutural striae deep and depressed near scutellum; intervals smooth, equal, convex, with a single row of fine punctures, wider than striae; humeri truncate, quadrate; tarsi longer than tibiae.

#### Description.

*Measurements* (male): **BL:** 3.29–3.40 mm; **EL:** 2.33–2.38 mm; **EWB:** 1.02–1.08 mm; **EWW:** 1.02–1.08 mm; **PL:** 0.96–1.02 mm; **PW:** 0.90–0.94 mm; **RL:** 0.52–0.55 mm; **RWA:** 0.31–0.34 mm; **RWB:** 0.25–0.30 mm; **AL:** 0.90–0.94 mm; **ASL:** 0.40–0.42 mm; **AFL:** 0.30–0.31 **ACL:** 0.20–0.22; **ACW:** 0.12–0.14 mm. *Measurements* (female): **BL:** 2.58–2.80 mm; **EL:** 1.70–1.90 mm; **EWB:** 0.84–0.96 mm; **EWW:** 0.84–0.96 mm; **PL:** 0.88–0.90 mm; **PW:** 0.77–0.78 mm; **RL:** 0.48–0.50 mm; **RWA:** 0.26–0.28 mm; **RWB:** 0.19–0.20 mm; **AL:** 0.70–0.90 mm; **ASL:** 0.32–0.40; **AFL:** 0.20–0.30; **ACL:** 0.13–0.20; **ACW:** 0.10–0.11 mm.

**Figures 1–4.**
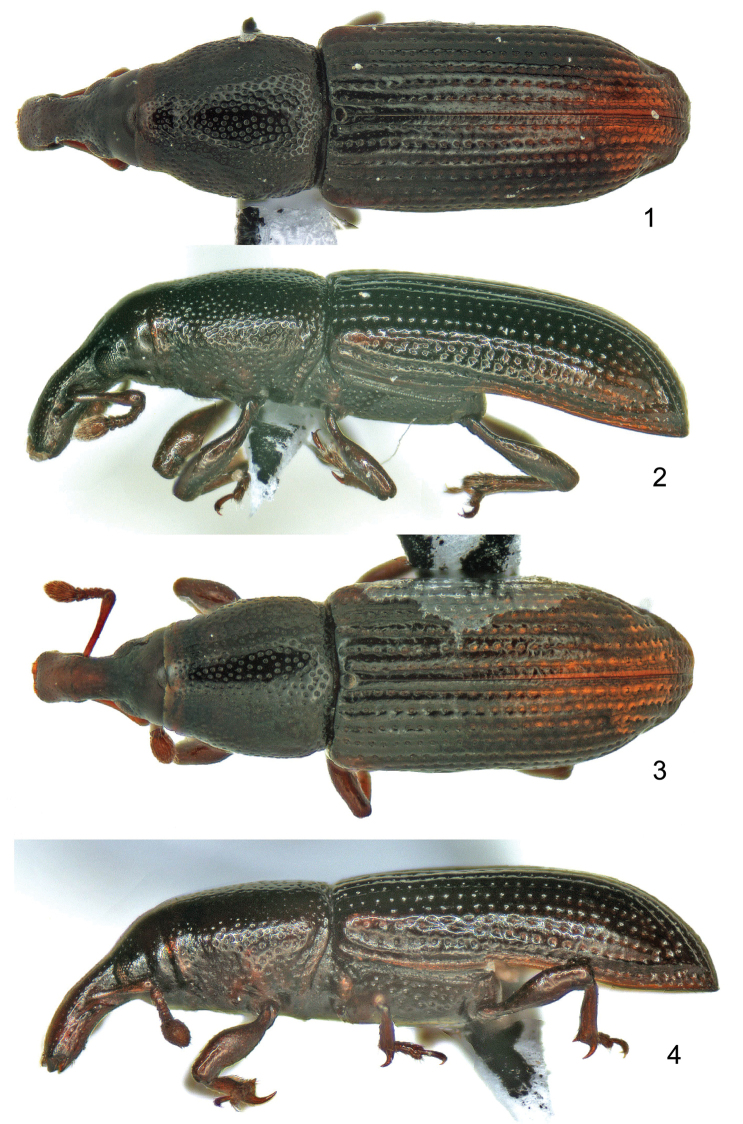
*Orthotemnus longitarsus* habitus. **1** ♂ dorsal aspect **2** ♂ lateral aspect **3** ♀ dorsal aspect **4** ♀ lateral aspect.

**Figures 5–11.**
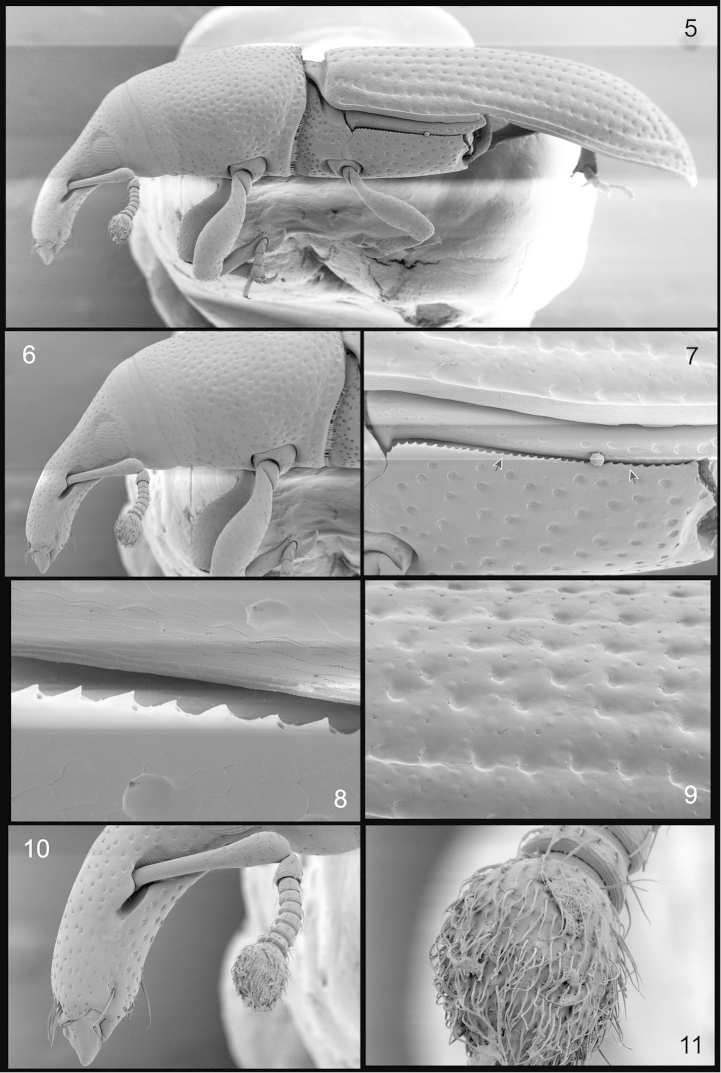
*Orthotemnus longitarsus* SEM photographs. **5** ♂ habitus, lateral aspect **6** enlargement of head and prothorax, lateral aspect **7** enlargement of lateral part of metathorax **8** enlargement of serrate dorsal margin of metaventrite **9** enlargement of left elytron **10** enlargement of apical half of rostrum, lateral aspect **11** enlargement of antennal club.

*Body* (Figs 1–5) oblong, curved, glossy.

*Colour* dark reddish-brown; rostrum, antennae and legs paler brown than body.

*Rostrum* (Figs 6, 10) long, more than 2 × longer than width at base, more or less 0.5 × as long as pronotum; narrow before antennal insertions (basal half), sides parallel, curved dorsally, widening after antennal insertion (in apical half), curved ventrally at antennal insertions; antennae inserted after middle of rostrum; with fine, subcircular, shallow punctures along dorsum, becoming oblong, denser towards apex; narrower at base than at apex; scrobes (Fig. 10). well-defined, deep, short, located in middle of rostrum, dorsal margin slightly lower than upper margin of eye, ventral margin black, visible in dorsal view.

*Mouthparts*. Maxillae (Fig. 25) with palpiger with large, slender seta near basolateral margin; galeo-lacinial complex with several large, tooth-like setae along margin, a few smaller, slender setae near base. Labium (Fig. 26) with prementum elongate, lacking setae; 1^st^ segment of palpus with 2 setae, 2^nd^ segment with 1 seta, 3^rd^ (apical) segment lacking setae. Right mandible (Fig. 24) with 3 teeth and a small molar region, primary tooth largest, second and third teeth smaller; left mandible (Fig. 23) mainly with large primary tooth and molar region, other teeth reduced to small ridges.

**Figures 12–18.**
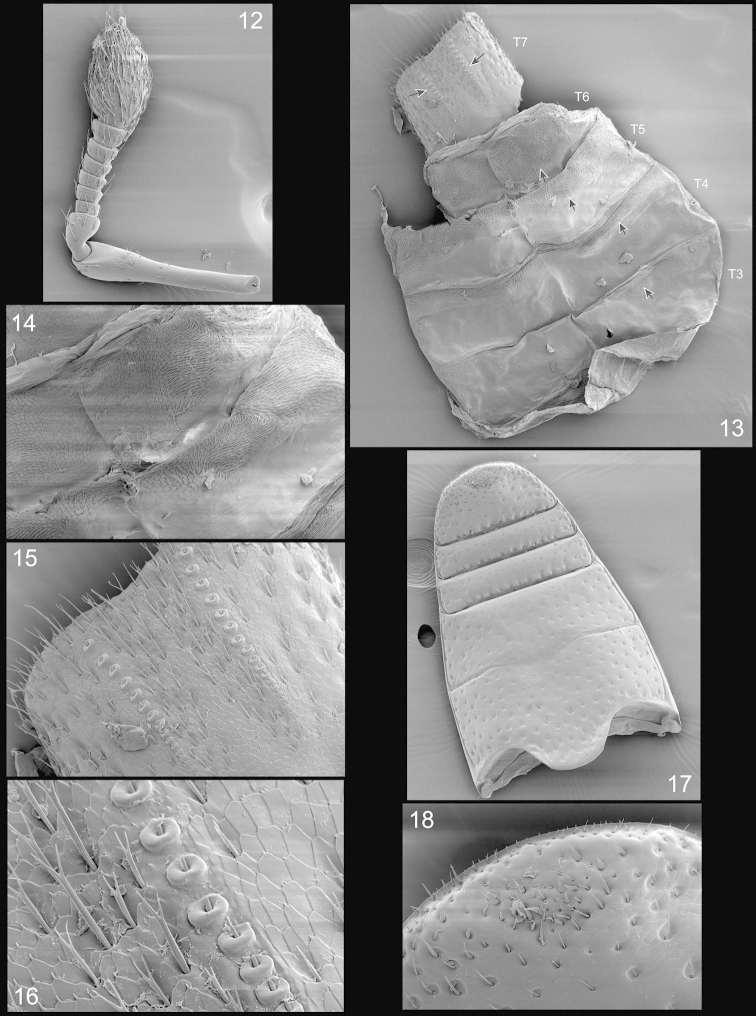
*Orthotemnus longitarsus* SEM photographs. **12** antenna **13** ♂ abdominal tergites **14** enlargement of portions of tergites 5 and 6 showing spiculate patches **15** enlargement of tergite 7 showing rows of plectra **16** enlargement of plectra on tergite 7 **17** ♂ abdominal venter **18** enlargement of apical portion of ventrite 5.

*Antennae* (Figs 10–12) pale, long, glossy brown, clubs pilose; scapes slightly bent at middle, thick and widening in distal third, widened part with sparse, shallow, oblong punctures with fine suberect setae, smooth, passing posterior margin of eye, approaching postocular constriction, approximately as long as or slightly shorter than funicle; clubs compact; funicles with all segments with few fine, erect setae, loose, segment 1 stout, longer than wide, segments 2–5 subequal, 6 and 7 wider than long, 7 wider than 6; clubs (Fig. 11). longer than wide, oval, slightly laterally flattened, 3-segmented, setigerous, setae suberect towards apex, acuminate.

*Head* oval, laterally constricted behind eyes, constriction weak dorsally, temples swollen, with subcircular punctures, fine sparse punctures behind postocular constriction, wrinkled, glossy; forehead slightly convex, broader than rostral base, with clear circular punctures, interspaces 2–3 × puncture diameter, with short median longitudinal furrow; vertex bulbous, convex, wrinkled. Eyes oval, widely separated, convex, with coarse, convex facets.

*Pronotum* conical, anteriorly constricted, deep laterally but weak dorsally, forming collar with large, circular punctures throughout, punctures separated by approximately 1–2 × their diameter, with abbreviated smooth median line not reaching anterior and posterior margins, dorsally and laterally convex, truncate along posterior margin.

*Mesonotum* (Fig. 29) typical of Cossoninae; axillary cord enlarged, lateral margins rounded.

*Scutellum* visible, transverse-oblong, smooth, dull.

*Metanotum* (Fig. 30) with metascutum reaching posterior margin of notum; scutellar groove nearly reaching posterior margin of notum; allocrista angular at anteromesal angle.

*Metendosternite* (Fig. 28) stalk slightly taller than wide; longitudinal flange short; furcal arm slender, bifid at apex; hemiductus slender.

*Proventriculus* as in Fig. 27.

*Elytra* (Figs 5, 9. glossy, long, disk convex, lateral margins parallel until declivity, base truncate, slightly broader than pronotal base; striae with large, deep circular punctures, punctures separated by approximately 1–1.5 × puncture diameter, first stria (sutural stria) deeper and depressed near scutellum; intervals smooth, equal, convex, with a single row of fine punctures, wider than striae; sutural interval deeper and depressed near scutellum, dilated at declivity to apex, interval 3 elevated caudally of declivity, with more than one row of fine punctures; intervals 4, 5 and 6 connected slightly caudally of declivity but not reaching apex; interval 9 forming oblique, ridged elevation caudally of declivity and connected to interval 3, not reaching apex; humeri truncate, quadrate.

*Hindwings* (Fig. 31) slender, lacking jugal area (anal lobe); *Rr* slender, abbreviated, not reaching *rcm*; *rc* absent; *1rs* triangular and larger than *2rs*; *R3* absent; *Cu1* not reaching posterior margin; *r-m* absent; *A* simple, other anal veins absent.

*Thoracic sterna* flat; prosternum wrinkled, with deep circular punctures; procoxae separated by 0.5 × diameter of coxa; mesoventrite flush with prosterum, base bisinuate, mesocoxae separated by approximately coxal diameter; metaventrite transverse, longitudinal sulcus from base to beyond middle, with circular punctures spaced by 2–3 × diameter of puncture, meta coxae separated by slightly less than 2 × diameter of coxa; dorsal margins of metaventrite serrate (sclerolepidial setae absent, though setal sockets present; Figs 7–8).

**Figures 19–22.**
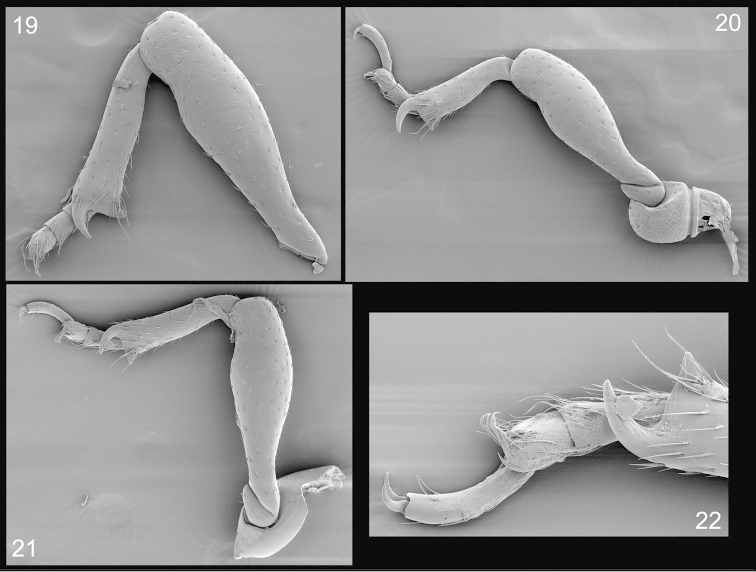
*Orthotemnus longitarsus* SEM photographs of legs. **19** foreleg **20** middle leg **21** hindleg **22** enlargement of hind tarsus.

*Legs* (Figs 19–22). Ffemora glossy, robust along distal two-thirds, with small, circular, sparse punctures, separated by approximately 2–4 × puncture diameter, slightly less than 2 × length of tibia; tibiae with smaller, similarly spaced punctures, straight, nearly uniform in width; uncus small, at outer apical margin, approximately 0.5 × as long as tarsomere 5, praemucro at inner apical margin covered by a small tuft of setae; tarsi longer than tibiae, 5-segmented; segments 1–3 with long, pale yellowish setae, 1 2 × longer than wide; 2 subequal in length and width; 3 wider than long, notched; 4 small, 5 longer than others combined, curved, dorsally convex, glossy, with appressed pale yellowish setae; claws simple, free.

**Figures 23–31.**
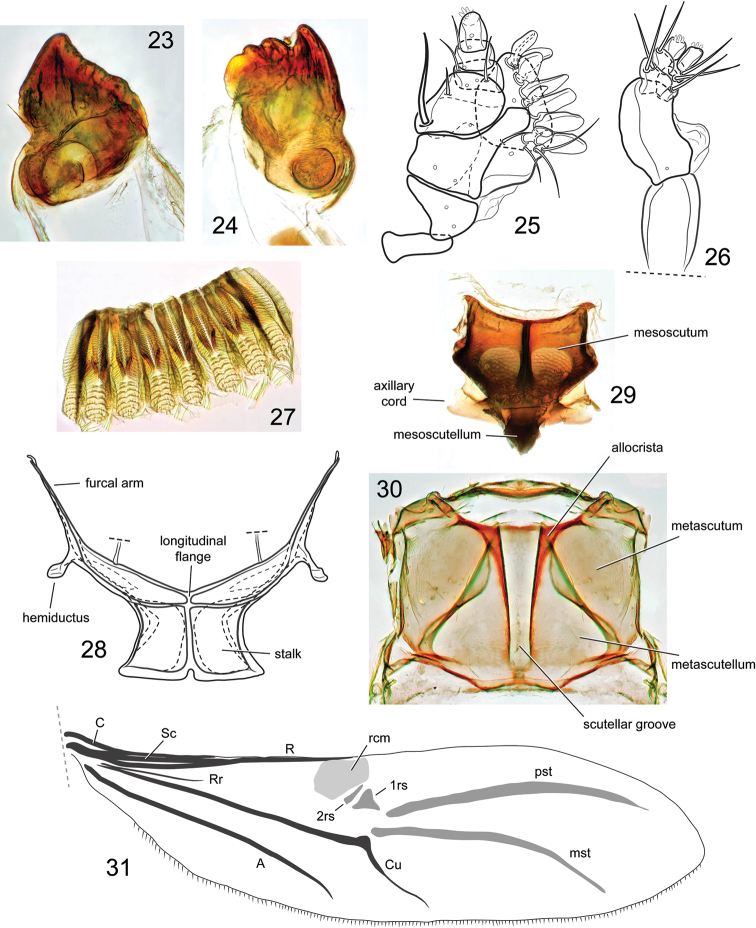
*Orthotemnus longitarsus* mouthparts, internal structures and hindwing. **23** left mandible **24** right mandible **25** maxilla **26** labium **27** proventriculus **28** metendosternite **29** mesonotum **30** metanotum **31** hindwing: **C** = Costa **Sc** = Subcosta **Rr** = radial recurrent vein **R** = Radius **rcm** = margin of radial cell **2rs, 1rs** = radial sclerites **pst** = postradial stripe **mst** = medial stripe **Cu** = Cubital **A** = Anal.

*Abdomen.* **Tergites** (Figs 13–16) with microtrichial wing-locking patches restricted to posterior half of tergites 2–5 and present on most of tergite 6; two linear rows of plectra present on tergite 7 (Lyal and King 1996). **Ventrites** (Fig. 17, 18). glossy, 1 and 2 with large, circular punctures, punctures separated by 2–3 × their diameter; 1 broader than 2, projecting between metacoxae, 1 and 2 clearly separated; 3 and 4 narrow, equal, each with two rows of punctures, basal row with large, deep, oblong punctures, apical row with fine small punctures, with fine suberect setae apically; 5 transverse, posterior margin rounded, with large, oblong, deep punctures at base, punctures becoming sparse and subcircular apically with some appressed setae.

**Figures 32–37.**
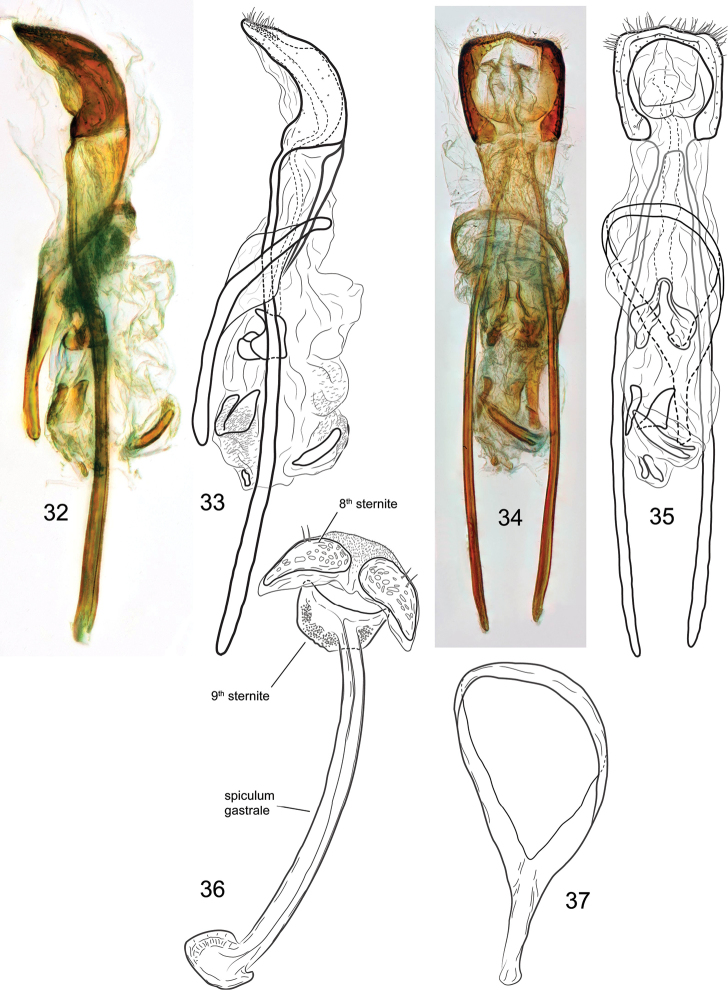
*Orthotemnus longitarsus* ♂ terminalia. **32–33** Penis, lateral aspect **34–35** aedeagus, dorsal aspect **36** sternites VIII and IX **37** tegmen.

*Male terminalia and genitalia* (Figs 32–37). Spiculum gastrale of sternite IX with flattened, expanded apex; apex approaching size of sternite at base. Sternite VIII divided, with 2 setae on each hemisternite. Tegmen complete; manubrium nearly 0.5 × length of tegmen. Penis with temones ca. 4.5 × body of penis; apical margin setose; endophallus (internal sac) extensive, greater than 0.5 × length of temones, bearing several internal sclerites from approximate middle of endophallus to its apex.

**Figures 38–40.**
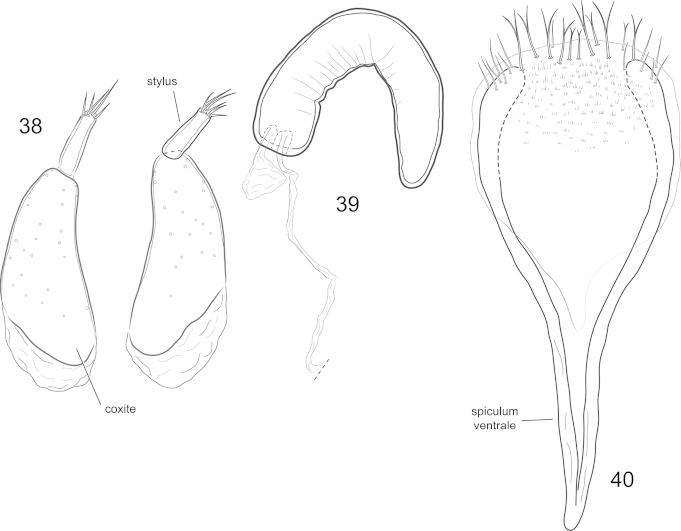
*Orthotemnus longitarsus* ♀ terminalia. **38** ovipositor (coxites and styli) **39** spermatheca **40** sternite VIII.

*Female terminalia and genitalia* (Figs 38–40). Gonocoxites of typical form; oblong; styli elongate, narrow. Spermatheca approximately crescent-shaped. Sternite VIII with spiculum ventrale gradually narrowing towards apex; base with many setae, setae mostly bifid at middle of base and mostly simple laterally; many microtrichia along basal region of sternite anteriorly of setae.

#### Material examined.

**Holotype:** male: China: Jiangsu: Jiangsu Province, Nanjing, Zijingshan, 32.30°N, 118.49°E, 05 III 2008, collected by Liang Ding (IOZ). **Paratypes:** 4 ♀ and 4 ♂: same data as holotype; found under trees in park.

#### Etymology.

The specific epithet is a combination of the Latin word *longus* and the Latinized Greek word *tarsus*, referring to the longer tarsus in relation to the tibia; it is a noun in apposition.

#### Distribution.

China: Jiangsu Province: Nanjing, Zijingshan.

#### Sexual dimorphism.

The female (Figs 3–4) is smaller than the male (Figs 1–2), and its rostrum is slightly shorter and slenderer than that of the male. Males also have concave first and second ventrites, the first slightly more projected between the metacoxae than in the female.

## Discussion

The new species here differs from other species of the genus as detailed in the key below as well as in the following features: its large size; the rostrum narrow and strongly convex, narrow basally after the antennal insertions but widening distally; the forehead with a short, median longitudinal furrow starting in the basal half and extending posteriad; the long scapes reaching the postocular constriction; the swollen temples; the transverse-oblong scutellum; the quadrate humeri; the parallel-sided tibiae; the tarsi being longer than the tibiae. This species marks the first record of the genus for China.

The genus is currently known to comprise six species, including the one newly described here, distributed as follows: *Orthotemnus reflexus* and *Orthotemnus disparilis* in the Australian, *Orthotemnus filiformis* and *Orthotemnus expansus* in the Afrotropical and *Orthotemnus ulmi* and the new species in the Palaearctic region (Csiki 1936, Alonso-Zarazaga and Lyal 1999, Setliff 2007). The genus has seemingly not been recorded from the Nearctic, Neotropical, nor Oriental regions. This distribution suggests that the genus may occur more widely in the Old World including the Oriental region.

### Key to the species of *Orthotemnus*

**Table:** 

1	Apical margin of elytra reflexed or recurved	**2**
–	Apical margin of elytra normal, subacute	**4**
2	Elytral intervals flat, obsoletely punctate; prothorax conical, strongly constricted before apex; closely, rather coarsely punctate, except along abbreviated narrow median space; interspaces finely alutaceous	***Orthotemnus filiformis***
–	Elytral intervals depressed or weakly convex, finely shagreened or sparsely and finely punctate; prothorax slightly constricted or with flat constriction not reaching disc at apex	**3**
3	Rostrum longer than pronotum, equal breadth throughout, with fine, dense, shallow punctures; prothorax elongate, triangular, with evenly sparse, moderately deep punctures; base truncate and as broad as elytra, weakly constricted at apex	***Orthotemnus reflexus***
–	Rostrum shorter than pronotum (4:5), conical, gradually narrowing from base to apex, with fine sparse punctures; short elongate fovea between antennal insertions; prothorax less elongate than in *Orthotemnus reflexus*; punctures round, shallow, rather dense, unevenly distributed; base weakly bisinuate, slightly narrower than elytra	***Orthotemnus ulmi***
4	Antennae inserted before middle of rostrum; rostrum strongly curved at middle or at point of antennal insertions	**5**
–	Antennae inserted behind middle of rostrum; rostrum slightly curved	***Orthotemnus expansus***
5	Prothorax small, oblong, with large coarse punctures; elytra short, gradually narrowing to apex; striae with large, dense, quadrate punctures	***Orthotemnus disparilis***
–	Prothorax conical, constricted anteriorly; constriction deep laterally and weak dorsally, with large, circular punctures throughout; elytra long, parallel-sided until declivity; striae with large, deep, circular punctures	***Orthotemnus longitarsus* sp. n.**

## Supplementary Material

XML Treatment for Orthotemnus

XML Treatment for Orthotemnus longitarsus
